# Early termination in single-parameter model phase II clinical trial designs using decreasingly informative priors

**DOI:** 10.18203/2349-3259.ijct20221110

**Published:** 2022-04-25

**Authors:** Chen Wang, Roy T. Sabo, Nitai D. Mukhopadhyay, Robert A. Perera

**Affiliations:** Department of Biostatistics, Virginia Commonwealth University, Richmond VA, U. S. A.

**Keywords:** Bayesian methods, Early termination, Hypothesis testing, Phase II clinical trial

## Abstract

**Background:**

To exchange the type of subjective Bayesian prior selection for assumptions more directly related to statistical decision making in clinician studies and trials, the decreasingly informative prior (DIP) is considered. We expand standard Bayesian early termination methods in one-parameter statistical models for Phase II clinical trials to include decreasingly informative priors (DIP). These priors are designed to reduce the chance of erroneously adapting trials too early by parameterize skepticism in an amount always equal to the unobserved sample size.

**Method:**

We show how to parameterize these priors based on effective prior sample size and provide examples for common single-parameter models, include Bernoulli, Poisson, and Gaussian distributions. We use a simulation study to search through possible values of total sample sizes and termination thresholds to find the smallest total sample size (N) under admissible designs, which we define as having at least 80% power and no greater than 5% type I error rate.

**Results:**

For Bernoulli, Poisson, and Gaussian distributions, the DIP approach requires fewer patients when admissible designs are achieved. In situations where type I error or power are not admissible, the DIP approach yields similar power and better-controlled type I error with comparable or fewer patients than other Bayesian priors by Thall and Simon.

**Conclusions:**

The DIP helps control type I error rates with comparable or fewer patients, especially for those instances when increased type I error rates arise from erroneous termination early in a trial.

## INTRODUCTION

Phase II clinical studies typically focus on determining whether a treatment has sufficient evidence of preliminary efficacy to warrant further investigation, such as in phase III trials, or whether the investigation should be discontinued due to a lack of efficacy or safety. These studies tend to be small, and data monitoring tends to occur as subjects are accrued so that decisions on whether to stop the study early-for efficacy, safety, or futility-can be made as soon as possible, even before the planned end of the study.

While the traditional frequentist methods (e.g., Pocock group sequential designs, O’Brien-Fleming alpha-spending function, etc.) provide stopping rules or termination guidance in phase II trials, Bayesian methods allow the inclusion of prior or historical information, which may help to improve decision making.^1,2^ The Bayesian approaches are also more amenable to adaptive designs and complex modelings.^3^ Despite these benefits, the Bayesian approach can be subject to inflated type I error rates.

Further, the prior selection in Bayesian approach is crucial because it is possible to generate posterior distributions that are strongly influenced by the priors which is not desirable. In practice, the Bayesian approach can be contentious when prior information is based mainly on subject matter experts.^3^ To exchange this type of subjectivity for assumptions more directly related to statistical decision making in clinician studies and trials, the DIP is considered, where null skepticism is elicited into the prior in a manner that decreases its prior effective sample size (ESS) as subjects accrued.^4–6^ In this way, the posterior distribution is increasingly informed by observed data and less by the prior information as subjects are accrued.

The goal of this paper is to develop and present the DIP approach based on the effective sample size for single-parameter models, include Bernoulli, Poisson, and Gaussian, and compare the DIP approach to the Thall and Simon’s Bayesian approaches.^1,2^ The net effect of this DIP formulation is that it restricts response-based adaptation early in a trial, gradually permitting more adaptation as the overall Bayesian model transfers the total effective sample size from the prior to the likelihood. If applied to designs featuring early termination processes, this decreasingly informative prior could possibly help control type I error rates, especially for those instances when increased type I error rates arise from erroneous termination early in a trial.

This paper presents an alternative Bayesian approach to early termination in phase II trials using DIP in single-parameter statistical models. Following a description of standard Bayesian early termination phase II trial designs in Section 2.1, the rationale of DIP approach and the general model is detailed in section 2.2. Examples of one-sample models, including Bernoulli, Poisson, and Gaussian distributions, are presented in 2.3. Simulation studies (Section 3) are used to compare the performance of the DIP approach with the standard Bayesian model, focusing on identifying admissible designs (those with at least 80% power and no more than 5% type-I error rates) and the minimum sample size that yields such designs. Section 4 concludes the paper with a discussion.

## METHODS

### Standard Bayesian early termination phase II trials

In single-group phase II studies and two-group phase II trials, we often need to know if an experimental treatment is sufficiently efficacious relative to some threshold or the other treatment. Suppose we have a likelihood function f(y|θ) and prior distribution π(θ) for outcome vector y and scalar parameter θ. Let θ_1_ be the parameter value representing efficacy in a new treatment, while θ_2_ reflects either some null level representing the boundary between an efficacious and non-efficacious treatment or the efficacy parameter in a comparison group. Then, the hypotheses we are testing are


$${\text{H}}_{0}:\;{\text{θ}}_{1}\leq {\text{θ}}_{2}+{\text{δ}}_{0},\;{\text{H}}_{1}:{\text{θ}}_{1}>{\text{θ}}_{2}+{\text{δ}}_{0}$$

where δ_0_ is a fixed targeted improvement for the new treatment to achieve (which could be 0). Note that these hypotheses assume that larger values of Θ_1_ are reflective of greater efficacy; we could simply switch the directions of the inequalities if lower values imply greater efficacy. We also set upper and a lower boundary for the posterior probability, denoted as p_s_ and p_f_ respectively, representing the probabilistic thresholds needed to be met in order to terminate the trial for superiority or futility. Throughout the trial, we can decide to terminate for efficacy if the evidence is promising (P(θ_1_>θ_2_+δ_0_|y)≥p_s_ or terminate for futility if the evidence is unpromising (P(θ_1_>θ_2_+δ_0_|y)≤p_f_), and we continue the trial and enrol additional subjects if the evidence is inconclusive (p_f_<P(θ_1_>θ_2_+δ_0_|y)<p_s_). These probabilities can be estimated and the resulting decisions can be made after each new subject is enrolled and observed until the new treatment is determined as either efficacious or futile, or when all the predetermined total number of subjects are recruited. Note that posterior probabilities could be calculated after cohorts of patients are accrued and observed, though we will not investigate that possibility here.

### Decreasingly informative prior

A DIP is a skeptical prior that decreases in ESS as a trial progress. To that end, it incorporates both the predetermined total sample size and the current observed sample size in such a way that the unobserved sample size N–n is made explicitly or approximately equal to the prior ESS in the prior distribution. The DIP is also parameterized in a way that centers the prior distribution at some value or values that would reflect conditions of the null hypothesis (i.e., the new therapy is not efficacious).

The basic steps for constructing a DIP are as follows: Determine the prior ESS for a statistical model, functionalize the prior in terms of the observed sample size n and the planned sample size N (often N–n, the unobserved sample size) so that the prior ESS is N at the beginning of the trial and 0 at the end of the trial, center the prior distribution at some value reflecting the null hypothesis, which could come from a hyperprior.

Though several approaches are available, ESS can be determined using the expected local-information-ratio approach.^7,8^ For example, given binary outcomes with response rate p and a prior beta (a and b), we know the mode of the prior is $\frac{\text{a}-1}{\text{a}+\text{b}-2}$, as well as the prior ESS= a+b. If we want the mode of prior centered around p_0_, the value from the null hypothesis, then we can set prior ESS=a+b=N–n and $\frac{\text{a}-1}{\text{a}+\text{b}-2}={\text{p}}_{\text{0}}$, and solve to get a=l+p_0_(N−n−2) and b=1+(1–p_0_)(N−n−2). We could slightly alter the prior parameterization of a and b as a=1+p_0_(N−n) and b=1+(1−p_0_)(N−n) so that the prior would be non-informative when the unobserved sample size is 0 at the end of the trial. Similarly, if we want the prior mean centered around a null value of p_0_, then we let prior ESS=a+b=N−n and $\frac{\text{a}}{\text{a}+\text{b}}={\text{p}}_{\text{0}}$. and derive a=p_0_(N−n) and b=(1−p_0_)(N−n); we again set a=1+p_0_(N−n) and b=1+(1−p_0_)(N−n) to make sure we have at least non-informative prior information when n=N in the trial.

In clinical trials, the number of accrued subjects n is small at the beginning of a trial relative to the planned sample size N, making the prior ESS large (e.g., 2+N−n) and the DIP informative. Since the prior is skeptical and parameterized to reflect the conditions stated in the null hypothesis (with mean or mode set to θ_0_), the resulting posterior distribution, with a low effective sample size n in the likelihood function, will be restricted from providing evidence in the form of posterior probabilities that would favor termination of the trial. As the trial progresses, and the prior ESS is “transferred” to the likelihood via the increased observed sample size, the posterior distribution becomes increasingly more sensitive to the likelihood and terminating the trial-if evidence for concluding as such is present-becomes more likely.

### Examples

#### Bernoulli data with a beta prior

Thall and Simon evaluate the efficacy of a new treatment based on Bernoulli outcomes where the interested parameter is the response rate.^1^ In this case, we assume θ=P and we have the likelihood distribution

$$\text{f}(\text{y}|\theta )=\text{f}(\text{y}|\text{p})=\prod_{\text{i}=1}^{\text{n}}{\text{p}}^{{\text{y}}_{\text{i}}}{\left(1-\text{p}\right)}^{1-{\text{y}}_{\text{i}}}.$$

In the one-sample case, we temporarily assume a non-informative prior distribution π(θ)=π(p) ~ beta (1,1); we will relax this assumption in subsequent paragraphs. Let θ=p denote the response rate of the new treatment and θ_0_=p_0_ denote the null response rate (which could be taken as the standard or current rate), we can derive the posterior distribution of p based on the conjugate nature of the beta-binomial pairing p|y ~ beta (1+y, 1+n−y), where $\text{y}=\sum_{\text{i}=1}^{\text{n}}{\text{y}}_{\text{i}}$ is the total number of successes out of the n observed subjects in the trial.

Instead of assuming a non-informative prior, we could elicit an informative prior-as suggested by Thall and Simon-by setting the prior mean equal to p_0_+δ_0_/2 and selecting a value for concentration parameter c_e._^1^ Thus, we can reparameterize the beta (a, b) prior using a=c_e_(p_0_+δ_0_/2) and b=c_e_[1−(p_0_+δ_0_/2)]. Thall and Simon discuss several possible values, including low values of c_e_ (e.g., 2) representing a sparse prior distribution, and larger values c_e_ (e.g., 10) representing an informative prior distribution localized around its mean.^1^ The posterior distribution of p for an informative prior is then given by p|y ~ beta (a+y, b+n−y).

With a binary outcome and conjugate beta prior distribution, an informative and skeptical DIP can be specified as beta (1+p_0_(N−n), 1+(1−p_0_)(N−n)), as discussed in Section 2.2. Combining the DIP with the binomial likelihood function, the posterior distribution of p is p|y ~ beta (1+ p_0_(N−n)+y, 1+(1−p_0_)(N−n)+(n−y)).

At the beginning of the trial, n and y are small and the posterior distribution of p is more centered at the prior mode p_0_. As n and y become larger, the accrued data become increasingly more important while the prior information is decreasing in importance.

#### Poisson data with a gamma prior

If the outcome in a clinical trial is the number of the events per subject, then a Poisson distribution with Gamma prior is a plausible choice for likelihood. One choice of the standard Bayesian prior distribution is a Jeffreys’ non-informative Gamma prior (limiting case) λ~Gamma (0.5, 0.001) and the posterior is λ|y ~ Gamma (0.5+y, 0.001+n). To apply a DIP for the one-sample case with Poisson outcomes with mean event rate θ = λ and a prior Gamma (a, b), we know the null mean (λ_0_) of the prior is $\frac{a}{b}$ and the prior ESS=b.^7^ If we want the prior centered around its null mean, then set prior ESS b=N−n and $\frac{\text{a}}{\text{b}}={\text{λ}}_{0}$, and get a=λ_0_(N−n) and b=N−n. Then, functionalize a and b as a=0.5+λ_0_(N−n) and b=0.001+(N−n) so that the prior would be non-informative when the unobserved sample size is 0 at the end of the trial. Thus, the DIP model for the count outcomes is defined as follows: y|λ ~ Poisson (λ)

$$\begin{array}{c} \text{λ}\sim \text{Gamma}\;\left(0.5+{\text{λ}}_{0}(\text{N}-\text{n}),\;0.001+(\text{N}-\text{n})\right)\\ \text{λ}|\text{y}\sim \text{Gamma}\;\left(0.5+{\text{λ}}_{0}(\text{N}-\text{n})+\text{y},\;0.001+\text{N}\right) \end{array}$$

In the DIP approach, when more subjects n is accrued in the trial, the skewness of the posterior distribution will depend more on the observed data instead of the prior information.

#### Normal data with known variance

For outcomes that could be modeled with a normal distribution with variance s^2^ known, we have the likelihood function f(y|θ) ~ N (θ, s^2^) with a normal prior θ ~ N (θ_0_, τ^2^). In this case, the prior ESS=s^2^/τ^2^.^7^ In a one-sample clinical trial, we assume θ=μ is the new treatment mean and τ^2^=s^2^/n_0_, where n_0_ is the prior ESS with a null-mean μ_0_. For the given likelihood y|μ ~ N (μ, s^2^) and prior μ ~ N (μ_0_, s^2^/n_0_), the posterior distribution of μ can be written as:

$$\text{μ}|{\text{s}}^{\text{2}},\text{y}\;\sim \;\text{N}\left(\frac{{\text{n}}_{\text{0}}}{{\text{n}}_{\text{0}}+\text{n}}{\text{μ}}_{\text{0}}+\frac{\text{n}}{{\text{n}}_{\text{0}}+\text{n}}\bar{\text{y}}\text{,}\frac{{\text{s}}^{\text{2}}}{{\text{n}}_{\text{0}}+\text{n}}\right)$$

The value of prior parameters n_0_ determines the level of information contained in the prior and the contribution of the null mean. If n_0_ is small and s^2^/n_0_ is large, the prior distribution is dispersed and less informative; when n_0_ is larger, the prior distribution will be more tightly centered around the null mean and become more informative.

For the DIP model, we set a skeptical prior (centered at μ_0_) as initially informative with n_0_=N−n. This formulation allows the information contained within the posterior distribution of μ to shift from the skeptical prior at the beginning of the trial to the likelihood function as subjects accrued. The DIP posterior distribution of μ is:

$$\text{μ}|{\text{s}}^{\text{2}},\text{y}\;\sim \;\text{N}\left(\frac{\text{N}-\text{n}}{\text{N}}{\text{μ}}_{\text{0}}+\frac{\text{n}}{\text{N}}\bar{\text{y}}\text{,}\frac{{\text{s}}^{\text{2}}}{\text{N}}\right)$$

As the posterior mean in the Bayesian model is a weighted average of the prior mean μ_0_ and the sample mean $\bar{y}$, this DIP formulation will cause the posterior mean to approximate the prior mean μ_0_ early in a trial and will increasingly approximate $\bar{y}$ as subjects accrued.

### Simulation templates

The goals of our simulation studies are to identify the smallest possible sample size N among admissible designs and to compare the DIP approach with other Bayesian approaches. We define an admissible design as having at least 80% power and no greater than 5% type I error rate. If there are no admissible designs based on power, we default to selecting the combination of parameters that yield the highest power and best-controlled type I error. If there are no admissible designs based on type I error, we default to selecting the combination of parameters that yield the lowest type I error and at least 80% power. We will explore Bernoulli-, Poisson- and normal-distributed outcomes.

In these simulations, the observed outcome y_i_ for each subject in each trial is randomly simulated from the probability density or mass function f (y_i_|θ), where θ is based off the population-level values assumed for that trial. Each subsequent subject is recruited until the trial is stopped (for futility or efficacy) or the planned sample size N is reached. In all one-sample cases, the upper (efficacy) and lower (futility) decision boundaries are set to p_s_ ∈ (0.80, 0.99) and p_f_ ∈ (0.01, 0.10) respectively, and the total sample size N∈(10, 100). For simplicity, we assume the target threshold δ_0_ equals 0. We simulate the observed data and estimate the power and type I error for each combination of p_s_, p_f_ and the planned total sample size N. We then select the smallest total sample size under the admissible power and type I error. Type I error is measured as the proportion of trials where the null hypothesis is rejected under the null hypothesis (e.g., θ_1_=θ_0_ for one-sample case), while power is measured as the proportion of trials where the null hypothesis is rejected under the alternative hypothesis (e.g., θ_1_>θ_0_). Each parameter setting is repeated in 1000 simulated trials. All simulations are coded using R 1.4.1717.^9^ The random samples are generated with the same seed.

For the Bernoulli outcome when θ=p, we assume the treatment group with higher response rate is more efficacious, and consider several models: a non-informative prior beta (1,1), informative prior beta (a, b) with several choices of prior information a+b=2, 6 or 10, and a DIP skeptical prior, as illustrated in section 2.3.1.

In the one-sample case, we consider null response rates of p_0_=0.1, 0.3, 0.5, or 0.7, with the actual response rate for the new treatment response rate p_1_ set at p_1_=p_0_+δ, where we range δ ∈ (0, 0.05, 0.10, 0.15, 0.2), while the target improvement is set at δ_0_=0 for simplicity. The outcome for each subject is randomly generated from Bernoulli (p_1_).

For the Poisson outcomes where θ=λ, we assume the lower values of event rates imply improved efficacy, and consider a Jeffrey’s non-informative prior (limiting case) gamma (0.5, 0.001) and a decreasingly informative prior (DIP) stated in section 2.3.2. We set the null event rate as λ_0_=0.5 or 5, and define the new treatment event rate as λ_1_=λ_0_-δ. When λ_0_=0.5, we set δ ∈ (0, 0.05, 0.10, 0.15, 0.2); when λ_0_=5, we set δ ∈ (0, 0.5, 1, 1.5, 2). Each subject is randomly generated from Poisson (λ_1_).

For the normal cases where θ=μ with known variance, we consider Bayesian models with n_0_=2, 6, or 10 for Equation 2, as well as a DIP case. We study low-variance and high-variance cases reflecting our assumptions about the known variability. For each template, we set the null mean as μ_0_=100 and expect lower values to imply improved efficacy; thus, the new treatment mean is defined as μ_1_=μ_0_-δ, where we set δ=0, 5, or 10 and set δ_0_=0 for simplicity; we consider the low variability with s=15 and the high variability with s=30. Each subject is randomly generated from N (μ_1_, s^2^).

## RESULTS

Table 1 shows the simulation results for one-sample Bernoulli cases with a low response rate (p_0_=0.1). Compared with different standard Bayesian approaches, the DIP approach always has better-controlled type I error, with a comparative or lower sample size. For some cases in which the standard Bayesian approach cannot achieve the admissible design on type I error, such as the case p_1_=0.2, the DIP approach not only controlled type I error, but also has the smallest planned sample size. Results are similar for other cases (p_0_=0.3, 0.5 and 0.7) (see Tables S.1, S.2, and S.3 in the supplementary material).

The simulation results for the one-sample Poisson cases are shown in Table 2. Compared to the non-informative Bayesian approach, the DIP approach performs better in controlling type I error. Additionally, when the effect size is large, the DIP approach has a lower sample size and better-controlled type I error rate.

Table 3 show the simulation results for one-sample normal cases. In both low and high variability settings, compared with different standard Bayesian approaches, the DIP approach has lower or comparative sample size when type I error is controlled (0.05). When the admissible type I error rate cannot be achieved, the DIP approach has a better-controlled type I error and comparable sample size.

## DISCUSSION

In summary, we introduced the rationale of the DIP approach, applied the DIP to three formulations of early termination phase II trial designs (Poisson, Bernoulli, and Normal), and compared the performance to the Bayesian approaches by Thall and Simon using simulation studies.^1,2^ The results show that, for the three distributions and across all one-sample settings, compared to the traditional Bayesian approaches by Thall and Simon, the DIP approach requires fewer patients when admissible designs are achieved.^1,2^ In the designs where type I error or power are not admissible, the DIP approach yields similar power and better-controlled type I error with comparable or fewer patients than Thall and Simon’s Bayesian approaches.^1,2^ We also extend the one-sample case to two-sample cases, and the results are presented in the supplemental material. For two-sample cases, it is concluded that the DIP approach performed better than Thall and Simon’s Bayesian approaches for moderate to large response rates, but performed poorly with low response rates and low effect sizes.^1,2^

It should be noted that the focus of this study is on identifying the smallest sample size to achieve an admissible design, defined by the commonly used thresholds of at least 80% power and at most 5% type-I-error. Changing the minimum power and maximum type I error rate might change our findings and conclusions, though these values are conventional. We also ignored admissible designs with a larger sample size, forfeiting designs with possibly higher power or lower type I error. While our choices for the predetermined sample size (N) are limited within the admissible design as having at least 80% power and no greater than 5% type I error, the choices for parameters settings in the simulations are broadly and comprehensively considered to reflect realistic scenarios. For each parameter set, we also investigated a non-informative and three informative models (κ_0_=2, 6 and 10) in comparison with the DIP model.

While we elicited the DIP in the way that is not based on any historical or optimistic prior, the researchers can still explore other subjective priors at the end of the trial to determine the robustness of their findings. We also motivated the DIP approach using conjugate examples: Poisson-gamma, beta-binomial, and normal-normal models. We can easily extend this to other prior-likelihood combinations, particularly those that lead to non-conjugate or intractable posterior distributions using MCMC approaches. The key of the DIP approach with a non-conjugate prior is to parameterize the prior so that its effective sample size equals N−n, which may require numerical or simulation-based determination.^7,8^ In future work, we plan to extend the single parameter DIP model to cases with two or more parameters.

## Supplementary Material

1

## Figures and Tables

**Table 1:** Simulation results for Bernoulli cases-one sample (p_0_=0.1).

Models	p_0_	p_1_	Sample size^a^	Futility	Efficacy	Power	Type I error^b^
**DIP**	0.1	0.15	94	0.01	0.89	0.808	0.239
**Bayesian (Beta (1, 1))**	0.1	0.15	96	0.02	0.94	0.802	0.294
**Bayesian (a+b=2)**	0.1	0.15	92	0.01	0.85	0.804	0.342
**Bayesian (a+b=6)**	0.1	0.15	100	0.01	0.87	0.810	0.295
**Bayesian (a+b=10)**	0.1	0.15	98	0.03	0.85	0.805	0.301
**DIP**	0.1	0.20	76	0.10	0.98	0.802	0.050
**Bayesian (Beta (1, 1))**	0.1	0.20	88	0.02	0.99	0.868	0.076
**Bayesian (a+b=2)**	0.1	0.20	85	0.01	0.99	0.816	0.050
**Bayesian (a+b=6)**	0.1	0.20	90	0.01	0.99	0.800	0.051
**Bayesian (a+b=10)**	0.1	0.20	86	0.07	0.98	0.820	0.050
**DIP**	0.1	0.25	42	0.06	0.98	0.843	0.050
**Bayesian (Beta (1, 1))**	0.1	0.25	38	0.03	0.99	0.811	0.056
**Bayesian (a+b=2)**	0.1	0.25	52	0.09	0.99	0.816	0.050
**Bayesian (a+b=6)**	0.1	0.25	43	0.01	0.97	0.816	0.050
**Bayesian (a+b=10)**	0.1	0.25	38	0.04	0.96	0.808	0.050
**DIP**	0.1	0.30	22	0.02	0.98	0.801	0.050
**Bayesian (Beta (1, 1))**	0.1	0.30	24	0.08	0.99	0.818	0.052
**Bayesian (a+b=2)**	0.1	0.30	25	0.08	0.97	0.821	0.050
**Bayesian (a+b=6)**	0.1	0.30	25	0.03	0.96	0.826	0.050
**Bayesian (a+b=10)**	0.1	0.30	25	0.08	0.95	0.810	0.050

a The planned sample size,

b Type I error is calculated under the null hypothesis p_1_=p_0_.

**Table 2:** Simulation results for Poisson cases.

Models	λ_0_	λ_1_	Sample size^a^	Futility	Efficacy	Power	Type I error^b^
**DIP**	0.5	0.45	100	0.10	0.80	0.695	0.424
**Bayesian**	0.5	0.45	98	0.02	0.80	0.793	0.587
**DIP**	0.5	0.4	99	0.03	0.87	0.804	0.253
**Bayesian**	0.5	0.4	80	0.05	0.90	0.807	0.355
**DIP**	0.5	0.35	98	0.02	0.96	0.803	0.075
**Bayesian**	0.5	0.35	97	0.06	0.97	0.831	0.138
**DIP**	0.5	0.3	68	0.07	0.98	0.806	0.050
**Bayesian**	0.5	0.3	86	0.06	0.99	0.845	0.058
**DIP**	5	4.5	98	0.03	0.96	0.802	0.071
**Bayesian**	5	4.5	99	0.09	0.97	0.802	0.147
**DIP**	5	4	29	0.03	0.97	0.808	0.050
**Bayesian**	5	4	37	0.02	0.99	0.802	0.050
**DIP**	5	3.5	12	0.09	0.96	0.819	0.050
**Bayesian**	5	3.5	14	0.03	0.97	0.832	0.050
**DIP**	5	3	10	0.03	0.95	0.945	0.050
**Bayesian**	5	3	10	0.04	0.96	0.931	0.050

a The planned sample size,

b type I error is calculated under the null hypothesis λ_1_=λ_0_.

**Table 3:** Simulation results for normal cases with known variance.

Models	μ_0_	μ_1_	s	Sample size^a^	Futility	Efficacy	Power	Type I error^b^
**DIP**	100	95	15	61	0.07	0.98	0.802	0.050
**Bayesian (κ_0_=2)**	100	95	15	71	0.03	0.99	0.814	0.050
**Bayesian (κ_0_=6)**	100	95	15	74	0.07	0.99	0.805	0.050
**Bayesian (κ_0_=10)**	100	95	15	67	0.02	0.98	0.808	0.050
**DIP**	100	90	15	19	0.06	0.97	0.869	0.050
**Bayesian (κ_0_=2)**	100	90	15	17	0.04	0.97	0.926	0.050
**Bayesian (κ_0_=6)**	100	90	15	16	0.06	0.95	0.816	0.050
**Bayesian (κ_0_=10)**	100	90	15	17	0.07	0.95	0.821	0.050
**DIP**	100	95	30	100	0.09	0.88	0.805	0.204
**Bayesian (κ_0_=2)**	100	95	30	98	0.08	0.92	0.802	0.272
**Bayesian (κ_0_=6)**	100	95	30	94	0.01	0.92	0.80	0.249
**Bayesian (κ_0_=10)**	100	95	30	98	0.05	0.91	0.803	0.246
**DIP**	100	90	30	60	0.05	0.97	0.811	0.050
**Bayesian (κ_0_=2)**	100	90	30	73	0.10	0.99	0.819	0.050
**Bayesian (κ_0_=6)**	100	90	30	73	0.01	0.99	0.805	0.050
**Bayesian (κ_0_=10)**	100	90	30	67	0.10	0.98	0.809	0.050

a The planned sample size,

b type I error is calculated under the null hypothesis μ1=μ0.
