# Supplementary material for: Early termination in single-parameter model phase II clinical trial designs using decreasingly informative priors
Source: Int J Clin Trials. Author manuscript; Available in PMC 2023 Feb 24. (PMC9957559; doi:10.18203/2349-3259.ijct20221110)
Supplement: 1 [file NIHMS1830181-supplement-1.pdf]

ANNEXURE

Table S.1: Simulation results for Bernoulli cases-one sample (p_0=0.3).

Model	p_0	p_1	Sample size[a]	Futility	Efficacy	Power	Type I error[b]
**DIP**	0.3	0.35	98	0.09	0.80	0.792	0.410
**Bayesian (Beta (1, 1))**	0.3	0.35	77	0.03	0.83	0.802	0.480
**Bayesian (a+b=2)**	0.3	0.35	98	0.02	0.83	0.800	0.453
**Bayesian (a+b=6)**	0.3	0.35	97	0.02	0.80	0.803	0.472
**Bayesian (a+b=10)**	0.3	0.35	96	0.04	0.81	0.809	0.460
**DIP**	0.3	0.40	100	0.03	0.95	0.804	0.097
**Bayesian (Beta (1, 1))**	0.3	0.40	98	0.07	0.97	0.806	0.174
**Bayesian (a+b=2)**	0.3	0.40	96	0.01	0.96	0.806	0.167
**Bayesian (a+b=6)**	0.3	0.40	100	0.07	0.95	0.802	0.156
**Bayesian (a+b=10)**	0.3	0.40	97	0.01	0.95	0.819	0.145
**DIP**	0.3	0.45	65	0.05	0.97	0.828	0.050
**Bayesian (Beta (1, 1))**	0.3	0.45	75	0.04	0.99	0.828	0.057
**Bayesian (a+b=2)**	0.3	0.45	72	0.02	0.99	0.813	0.052
**Bayesian (a+b=6)**	0.3	0.45	79	0.06	0.99	0.812	0.050
**Bayesian (a+b=10)**	0.3	0.45	76	0.03	0.98	0.830	0.050
**DIP**	0.3	0.50	36	0.07	0.97	0.808	0.050
**Bayesian (Beta (1, 1))**	0.3	0.50	44	0.05	0.99	0.819	0.050
**Bayesian (a+b=2)**	0.3	0.50	47	0.02	0.99	0.812	0.050
**Bayesian (a+b=6)**	0.3	0.50	48	0.07	0.99	0.806	0.050
**Bayesian (a+b=10)**	0.3	0.50	40	0.05	0.97	0.805	0.050

[a]The planned sample size, [b]type I error is calculated under the null hypothesis p1=p0.

Table S.2: Simulation results for Bernoulli cases-one sample (p_0=0.5).

Models	p_0	p_1	Sample size[a]	Futility	Efficacy	Power	Type I error[b]
**DIP**	0.5	0.55	94	0.08	0.80	0.767	0.399
**Bayesian (Beta (1, 1))**	0.5	0.55	96	0.05	0.82	0.802	0.480
**Bayesian (a+b=2)**	0.5	0.55	84	0.03	0.82	0.800	0.499
**Bayesian (a+b=6)**	0.5	0.55	95	0.03	0.83	0.801	0.450
**Bayesian (a+b=10)**	0.5	0.55	92	0.04	0.81	0.800	0.486
**DIP**	0.5	0.60	98	0.06	0.93	0.806	0.124
**Bayesian (Beta (1, 1))**	0.5	0.60	97	0.02	0.96	0.816	0.189
**Bayesian (a+b=2)**	0.5	0.60	98	0.03	0.96	0.809	0.196
**Bayesian (a+b=6)**	0.5	0.60	97	0.07	0.95	0.803	0.178
**Bayesian (a+b=10)**	0.5	0.60	90	0.01	0.94	0.800	0.169
**DIP**	0.5	0.65	68	0.04	0.97	0.810	0.050
**Bayesian (Beta (1, 1))**	0.5	0.65	82	0.09	0.99	0.806	0.056
**Bayesian (a+b=2)**	0.5	0.65	74	0.06	0.99	0.811	0.056
**Bayesian (a+b=6)**	0.5	0.65	79	0.02	0.99	0.812	0.051
**Bayesian (a+b=10)**	0.5	0.65	87	0.09	0.99	0.805	0.050
**DIP**	0.5	0.70	36	0.07	0.96	0.804	0.050
**Bayesian (Beta (1, 1))**	0.5	0.70	48	0.02	0.99	0.819	0.050
**Bayesian (a+b=2)**	0.5	0.70	48	0.04	0.99	0.830	0.051
**Bayesian (a+b=6)**	0.5	0.70	49	0.08	0.99	0.812	0.050
**Bayesian (a+b=10)**	0.5	0.70	45	0.04	0.98	0.822	0.050

[a]The planned sample size, [b]type I error is calculated under the null hypothesis p1=p0.

Table S.3: Simulation results for Bernoulli cases-one sample (p_0=0.7).

Models	p_0	p_1	Sample size[a]	Futility	Efficacy	Power	Type I error[b]
**DIP**	0.7	0.75	100	0.06	0.80	0.769	0.373
**Bayesian (Beta (1, 1))**	0.7	0.75	81	0.05	0.82	0.804	0.443
**Bayesian (a+b=2)**	0.7	0.75	98	0.02	0.88	0.803	0.450
**Bayesian (a+b=6)**	0.7	0.75	88	0.09	0.85	0.804	0.459
**Bayesian (a+b=10)**	0.7	0.75	99	0.02	0.85	0.801	0.434

Continued.

Models	p_0	p_1	Sample size[a]	Futility	Efficacy	Power	Type I error[b]
**DIP**	0.7	0.80	100	0.04	0.95	0.815	0.075
**Bayesian (Beta (1, 1))**	0.7	0.80	97	0.05	0.97	0.808	0.131
**Bayesian (a+b=2)**	0.7	0.80	98	0.05	0.98	0.800	0.148
**Bayesian (a+b=6)**	0.7	0.80	100	0.05	0.98	0.803	0.124
**Bayesian (a+b=10)**	0.7	0.80	94	0.05	0.97	0.803	0.114
**DIP**	0.7	0.85	50	0.07	0.96	0.816	0.050
**Bayesian (Beta (1, 1))**	0.7	0.85	70	0.01	0.99	0.850	0.050
**Bayesian (a+b=2)**	0.7	0.85	62	0.09	0.99	0.859	0.073
**Bayesian (a+b=6)**	0.7	0.85	63	0.02	0.99	0.834	0.050
**Bayesian (a+b=10)**	0.7	0.85	66	0.01	0.99	0.816	0.050
**DIP**	0.7	0.90	24	0.06	0.95	0.823	0.050
**Bayesian (Beta (1, 1))**	0.7	0.90	37	0.08	0.99	0.830	0.050
**Bayesian (a+b=2)**	0.7	0.90	27	0.10	0.99	0.802	0.050
**Bayesian (a+b=6)**	0.7	0.90	38	0.03	0.99	0.851	0.050
**Bayesian (a+b=10)**	0.7	0.90	29	0.10	0.97	0.809	0.050

[a]The planned sample size, [b]type I error is calculated under the null hypothesis p1=p0.

Table S.4: Simulation results for Bernoulli cases-two samples (p_2=0.1).

Models	p_1	p_2	Sample size[a]	Futility	Efficacy	Power	Type I error[b]
**DIP**	0.15	0.1	200	0.04	0.80	0.724	0.406
**Bayesian (Beta (1, 1))**	0.15	0.1	184	0.10	0.81	0.806	0.441
**Bayesian (a+b=2)**	0.15	0.1	194	0.01	0.84	0.804	0.429
**Bayesian (a+b=6)**	0.15	0.1	187	0.05	0.80	0.810	0.437
**Bayesian (a+b=10)**	0.15	0.1	192	0.04	0.80	0.807	0.422
**DIP**	0.20	0.1	197	0.07	0.87	0.817	0.260
**Bayesian (Beta (1, 1))**	0.20	0.1	199	0.03	0.95	0.801	0.158
**Bayesian (a+b=2)**	0.20	0.1	185	0.01	0.95	0.804	0.151
**Bayesian (a+b=6)**	0.20	0.1	198	0.01	0.94	0.800	0.133
**Bayesian (a+b=10)**	0.20	0.1	179	0.05	0.92	0.801	0.129
**DIP**	0.25	0.1	189	0.04	0.96	0.800	0.085
**Bayesian (Beta (1, 1))**	0.25	0.1	179	0.06	0.99	0.816	0.050
**Bayesian (a+b=2)**	0.25	0.1	184	0.08	0.99	0.805	0.050
**Bayesian (a+b=6)**	0.25	0.1	144	0.01	0.97	0.805	0.050
**Bayesian (a+b=10)**	0.25	0.1	156	0.08	0.96	0.811	0.050
**DIP**	0.30	0.1	147	0.03	0.98	0.802	0.050
**Bayesian (Beta (1, 1))**	0.30	0.1	91	0.09	0.97	0.805	0.050
**Bayesian (a+b=2)**	0.30	0.1	86	0.06	0.97	0.800	0.050
**Bayesian (a+b=6)**	0.30	0.1	88	0.08	0.95	0.809	0.050
**Bayesian (a+b=10)**	0.30	0.1	83	0.08	0.93	0.812	0.050

[a]The planned sample size, [b]type I error is calculated under the null hypothesis p1=p2.

Table S.5: Simulation results for Bernoulli cases-two samples (p_2=0.3).

Models	p_1	p_2	Sample size[a]	Futility	Efficacy	Power	Type I error[b]
**DIP**	0.35	0.3	191	0.03	0.80	0.670	0.383
**Bayesian (Beta (1, 1))**	0.35	0.3	178	0.01	0.82	0.802	0.569
**Bayesian (a+b=2)**	0.35	0.3	196	0.04	0.81	0.804	0.580
**Bayesian (a+b=6)**	0.35	0.3	193	0.04	0.80	0.800	0.563
**Bayesian (a+b=10)**	0.35	0.3	200	0.02	0.80	0.796	0.539
**DIP**	0.40	0.3	188	0.02	0.83	0.805	0.298
**Bayesian (Beta (1, 1))**	0.40	0.3	197	0.03	0.92	0.801	0.347
**Bayesian (a+b=2)**	0.40	0.3	196	0.02	0.92	0.803	0.341
**Bayesian (a+b=6)**	0.40	0.3	192	0.05	0.90	0.800	0.335
**Bayesian (a+b=10)**	0.40	0.3	189	0.03	0.89	0.805	0.325

Continued.

Models	p_1	p_2	Sample size[a]	Futility	Efficacy	Power	Type I error[b]
**DIP**	0.45	0.3	196	0.06	0.93	0.804	0.111
**Bayesian (Beta (1, 1))**	0.45	0.3	199	0.06	0.97	0.807	0.146
**Bayesian (a+b=2)**	0.45	0.3	190	0.04	0.97	0.807	0.162
**Bayesian (a+b=6)**	0.45	0.3	185	0.01	0.96	0.802	0.136
**Bayesian (a+b=10)**	0.45	0.3	198	0.02	0.96	0.803	0.125
**DIP**	0.50	0.3	168	0.06	0.97	0.814	0.050
**Bayesian (Beta (1, 1))**	0.50	0.3	192	0.03	0.99	0.859	0.061
**Bayesian (a+b=2)**	0.50	0.3	197	0.07	0.99	0.841	0.053
**Bayesian (a+b=6)**	0.50	0.3	178	0.02	0.99	0.800	0.050
**Bayesian (a+b=10)**	0.50	0.3	198	0.06	0.99	0.819	0.052

[a]The planned sample size, [b]type I error is calculated under the null hypothesis p1=p2.

Table S.6: Simulation results for Bernoulli cases-two samples (p_2=0.5).

Models	p_1	p_2	Sample size[a]	Futility	Efficacy	Power	Type I error[b]
**DIP**	0.55	0.5	199	0.02	0.80	0.651	0.380
**Bayesian (Beta (1, 1))**	0.55	0.5	200	0.01	0.83	0.805	0.589
**Bayesian (a+b=2)**	0.55	0.5	191	0.01	0.82	0.802	0.604
**Bayesian (a+b=6)**	0.55	0.5	196	0.02	0.80	0.811	0.585
**Bayesian (a+b=10)**	0.55	0.5	196	0.02	0.80	0.787	0.572
**DIP**	0.60	0.5	174	0.09	0.82	0.809	0.319
**Bayesian (Beta (1, 1))**	0.60	0.5	196	0.03	0.91	0.804	0.390
**Bayesian (a+b=2)**	0.60	0.5	190	0.05	0.90	0.804	0.402
**Bayesian (a+b=6)**	0.60	0.5	187	0.05	0.89	0.819	0.374
**Bayesian (a+b=10)**	0.60	0.5	194	0.01	0.89	0.808	0.342
**DIP**	0.65	0.5	190	0.05	0.92	0.807	0.115
**Bayesian (Beta (1, 1))**	0.65	0.5	193	0.01	0.97	0.809	0.182
**Bayesian (a+b=2)**	0.65	0.5	193	0.02	0.97	0.800	0.181
**Bayesian (a+b=6)**	0.65	0.5	195	0.02	0.96	0.802	0.148
**Bayesian (a+b=10)**	0.65	0.5	197	0.02	0.96	0.803	0.139
**DIP**	0.70	0.5	166	0.04	0.96	0.810	0.050
**Bayesian (Beta (1, 1))**	0.70	0.5	168	0.01	0.99	0.823	0.066
**Bayesian (a+b=2)**	0.70	0.5	166	0.02	0.99	0.810	0.071
**Bayesian (a+b=6)**	0.70	0.5	175	0.01	0.99	0.802	0.050
**Bayesian (a+b=10)**	0.70	0.5	193	0.05	0.99	0.800	0.051

[a]The planned sample size, [b]type I error is calculated under the null hypothesis p1=p2.

Table S.7: Simulation results for Bernoulli cases-two samples (p_2=0.7).

Model	p_1	p_2	Sample size[a]	Futility	Efficacy	Power	Type I error[b]
**DIP**	0.75	0.7	193	0.10	0.80	0.674	0.388
**Bayesian (Beta (1, 1))**	0.75	0.7	199	0.03	0.82	0.805	0.573
**Bayesian (a+b=2)**	0.75	0.7	192	0.02	0.83	0.811	0.584
**Bayesian (a+b=6)**	0.75	0.7	188	0.02	0.80	0.801	0.566
**Bayesian (a+b=10)**	0.75	0.7	197	0.01	0.80	0.795	0.557
**DIP**	0.80	0.7	187	0.02	0.84	0.826	0.281
**Bayesian (Beta (1, 1))**	0.80	0.7	188	0.01	0.93	0.803	0.329
**Bayesian (a+b=2)**	0.80	0.7	184	0.01	0.93	0.811	0.322
**Bayesian (a+b=6)**	0.80	0.7	193	0.03	0.92	0.807	0.308
**Bayesian (a+b=10)**	0.80	0.7	191	0.02	0.90	0.800	0.281
**DIP**	0.85	0.7	183	0.10	0.94	0.806	0.087
**Bayesian (Beta (1, 1))**	0.85	0.7	189	0.06	0.98	0.805	0.110
**Bayesian (a+b=2)**	0.85	0.7	172	0.08	0.98	0.802	0.119
**Bayesian (a+b=6)**	0.85	0.7	187	0.04	0.98	0.803	0.091
**Bayesian (a+b=10)**	0.85	0.7	191	0.10	0.97	0.808	0.087

Continued.

Model	p_1	p_2	Sample size[a]	Futility	Efficacy	Power	Type I error[b]
**DIP**	0.90	0.7	129	0.02	0.96	0.828	0.050
**Bayesian (Beta (1, 1))**	0.90	0.7	121	0.03	0.99	0.817	0.051
**Bayesian (a+b=2)**	0.90	0.7	124	0.09	0.99	0.820	0.053
**Bayesian (a+b=6)**	0.90	0.7	128	0.04	0.99	0.804	0.050
**Bayesian (a+b=10)**	0.90	0.7	140	0.05	0.98	0.857	0.050

[a]The planned sample size, [b]type I error is calculated under the null hypothesis p1=p2.
